# Accuracy of Body Mass Index and Obesity Status in Police Trainees

**DOI:** 10.3390/ejihpe12010004

**Published:** 2022-01-10

**Authors:** Miloš Stojković, Katie M. Heinrich, Aleksandar Čvorović, Velimir Jeknić, Gianpiero Greco, Filip Kukić

**Affiliations:** 1 Faculty of Sport and Physical Education, University of Belgrade, 1040 Belgrade, Serbia; milos_20175007@studentmail.fsfv.bg.ac.rs (M.S.); velimir_20165003@studentmail.fsfv.bg.ac.rs (V.J.); 2 Department of Kinesiology, Kansas State University, Manhattan, KS 66502, USA; kmhphd@ksu.edu; 3 Police Sports Education Center, Abu Dhabi Police, Abu Dhabi 253, United Arab Emirates; a.cvorovic@adpolice.gov.ae (A.Č.); f.kukic@adpolice.gov.ae (F.K.); 4 Department of Basic Medical Sciences, Neuroscience and Sense Organs, University of Study of Bari, 70121 Bari, Italy

**Keywords:** tactical athletes, health status, assessment, body fat, muscle mass

## Abstract

The first aim of this study was to compare body mass index (BMI) (indirect method) classification with the body fat percent (PBF) (direct method) and to determine how BMI classifies subjects with different levels of skeletal muscle mass percent (PSMM). The second aim was to determine the prevalence of overweight and obesity status among police trainees (PTs). A total of 103 male PTs participated in this research: age = 21.46 ± 0.64 years, body mass (BM) = 75.97 ± 8.10 kg, body height (BH) = 174.07 ± 6.31 cm, BMI = 25.05 ± 2.12 kg/m^2^. The InBody 370 multichannel bioelectrical impedance analysis (BIA) measured body composition. Study results indicated that muscular PTs could be misclassified as overweight and that PBF identified more subjects as obese. Namely, three PTs were obese according to BMI, while 13 were obese according to PBF. The information provided by this research could be used to help professionals understand the importance of measuring body composition, and the inaccuracies in BMI classification. In conclusion, whenever possible PSMM and PBF should replace the utilization of BMI to screen overweight and obesity in PTs. Agencies may think of using BIA as non-invasive, quick and inexpensive measurement tool.

## 1. Introduction

Body composition assessment is often used for members of the police force as an important tool for evaluation of physical fitness and health status [1,2,3]. Body composition is a valuable assessment since it is the result of various factors such as diet, stress, the amount of physical activity, and other daily habits [4,5,6,7]. Therefore, regular screening of body composition may provide a timely information on changes produced by these factors. To that end, bioelectrical impedance analysis has been often used as the simplest, most reproducible and least expensive method for body composition evaluation and has been most commonly used in practice by police forces as well as in the general population [8,9].

Body mass index (BMI) levels have been frequently correlated with physical fitness and used to evaluate body composition status in the police workforce [1,10,11,12,13]. Although BMI is the most frequently used method to assess body composition status, this method has been criticized because BMI does not always reflect true body fatness and has some limitations in assessing people with low muscle and high body fat, where these individuals have increased body fat and normal BMI [2,8,11]. Muscular individuals can also be misclassified as overweight or obese instead of normal weight [14,15]. This is problematic, since muscular police trainees (PTs) can be mistakenly classified with a group of obese individuals, whose physical training ability can be far below the more muscular PTs’ physical capabilities. This happens because BMI is exclusively based on height and weight calculations, while percent of body fat (PBF) more accurately shows level of obesity [16]. In addition, increased PBF impairs the level of physical abilities, while increased percent of skeletal muscle mass (PSMM) improves physical abilities, which is beneficial for performing professional duties, quality of life and general health in police forces [17,18]. As time spent in service is associated with increasing obesity [19], maintaining sufficient PSMM is both primary prevention and beneficial for physical performance [20,21,22,23]. In addition to BMI, PBF and PSMM, waist circumference (WC) is also a recommended measure of central adiposity (i.e., excess abdominal fat tissue). Increased WC indicates increased cardiovascular disease risk and it has been commonly used in body composition measurements for police forces [4,10,13].

Several studies have shown discrepancies between BMI, PBF and PSMM in assessing obesity among police forces [2,11,16]. Heinrich et al. [2] assessed the accuracy of BMI classification in relation to PBF among Russian POs. Their study found that BMI underestimated obesity rates when compared with PBF, and direct measurement of PBF could be more helpful than using estimation methods such as BMI. Similarly, Kukić et al. [11] compared whether the BMI misclassified the obesity rate of POs when compared to PBF, and the conclusion was also that BMI underestimated obesity among POs. They also examined the accuracy of BMI in evaluating obesity and found a moderate prediction value as the classification accuracy was 44.5–71.8%, depending on the age of the POs. Alasagheirin et al. [16] investigated the accuracy of BMI in estimating obesity compared to PBF among community police officers (POs). Their conclusion was that BMI underestimated the true prevalence of obesity and could represent a missed opportunity for early intervention and disease prevention.

Accurate evaluation of obesity and body composition assessment in PTs is highly important, especially for early identification and prevention of obesity, in order to be healthier and more productive at work once they become POs. Accordingly, the first aim of this study was to compare BMI classification as an indirect method of measuring obesity to the direct method of PBF and to determine how BMI classifies subjects who possess different levels of PSMM. The second aim was to determine the prevalence of overweight and obesity status among PTs.

## 2. Materials and Methods

This study was conducted as non-experimental cross-sectional study, using a random sample of PTs. The measurements were conducted in February 2019. The research was carried out in accordance with the conditions of the declaration of Helsinki: Ethical principles for medical research involving human subjects [24] and with the permission of the Ethics Committee of the ethical board of the Faculty of Sport and Physical Education, University of Belgrade (number 02-821/19-1).

### 2.1. Sample

The research was conducted on 103 male Police College trainees, with average age of 21.46 ± 0.64 years, body mass (BM) of 75.97 ± 8.10 kg, body height (BH) of 174.07 ± 6.31 cm and BMI of 25.05 ± 2.12 kg/m^2^. The sample size was provided by the academy management. All PTs in this research were in their final year of police studies, and they were all informed about the research before they agreed to participate in the study by providing oral informed consent.

### 2.2. Procedures

The anthropometric and body composition measurements were conducted early in the morning, before breakfast. Subjects were instructed not to consume food or drink before the testing and to have an adequate sleep, minimum seven hours prior to testing. They were all participating in physical training program at the police college campus, twice a day for five consecutive days a week, followed by a two-day rest period (weekend). Body composition was assessed using a multichannel bioelectrical impedance analysis in a laboratory setting, the InBody 370 machine (InBody, Seoul, South Korea), following previously established procedures [11,25]. The Bioelectrical Impedance Analysis (BIA) method is based on the fact that the human body consists of conductors and non-conductors. Generally, 50~70% of the human body consists of water which functions as a conductor, whereas body fat functions as a non-conductor [26]. The BIA method measures the impedance of the whole body on the assumption that the human body can be considered a cylinder and if *A* is the cross-sectional area, and *L* is the length, the impedance of the cylinder can be expressed in the following formula:

$$Z=p\;\frac{L}{A};\;p=\mathrm{resistivity}$$

On the other hand, if both sides are multiplied by *L*, we obtain the expression below. If we know the *L* and the impedance value, we obtain the volume. That is to say, if we know the height of the human body (acting as a conductor), and know the impedance value, we can obtain the volume of body water. Here, the volume represents an examinee’s height. Therefore, the two directly used variables in body composition analysis are impedance and height [26].

$$V=P\;{\frac{L}{Z}}^{2}(V\;\left(volume=A\;\left(area\right)\times L\;\left(length\right)\right)$$

The principle of the InBody370’s body composition analysis is explained by the following: the volume of body water, an electrolyte, is calculated first with a measured impedance value. Then, the value of lean body mass is calculated using the volume of body water. Body fat mass is determined by deducting the lean body mass from the measured weight. Height was entered by the user after being measured as described below. BM was also directly measured using the InBody370 [26]. 

Overall, the following data were obtained: BM, BH, BMI, skeletal muscle mass (SMM), PSMM, body fat (BF) and PBF. Body height (BH) was measured using the SECA 769 digital scale. The PTs were wearing sports shorts without a T-shirt, were barefoot, and had all metal, plastic, and magnetic accessories removed. They stood on the device and on the metal spots designated for their feet, while holding on to the lower and the upper edge of the handles. For BMI classification, categories from the World Health Organization (WHO) [27] were used: 18.5 to 24.9 kg/m^2^ for normal weight individuals, 25 to 29.9 kg/m^2^ for overweight, and ≥30 kg/m^2^ for obese. PBF was classified according to the American College of Sports Medicine [28] as essential fat (2.00–5.99%), skinny and athletes (6.00–13.99%), fitness (14.00–17.99%), average (18.00–24.99%) and obese (≥25%). PSMM was classified according to procedures explained in previous publications [29] as insufficient (<41.07), below average (41.08–45.41), average (45.42–49.75), above average (49.76–54.10) and excellent (≥54.11). 

### 2.3. Statistical Analysis

The data were analyzed descriptively using basic mean, standard deviations (SD), minimums, maximums, and coefficient of variation. Descriptive statistics for BMI, PSMM and PBF were determined using frequency analysis. The Kolmogorov–Smirnov test (K-S Test) was used to test the normality of distribution for all variables. To test the observed distribution of PBF and PSMM categories within BMI categories, crosstabs chi-square was employed. Pearson’s correlation coefficients were used as a measure of the association between BMI—PBF and BMI—PSMM. The effect sizes were described as ±0.2 small, ±0.5 medium and ±0.8 large [30]. The significance was set at *p* < 0.05. All analyses were conducted using the Statistical Program for Social Sciences (IBM, SPSS Statistics 26).

## 3. Results

The descriptive statistics for the sample age and body composition characteristics are shown in Table 1 as mean, SD, minimum and maximum values. All data were normally distributed except for BMI, where the Kolmogorov–Smirnov test was statistically significant (*p* = 0.002).

Regarding the BMI, PSMM and PBF classifications, frequency analysis was used as shown in Table 2. Overall, three subjects were obese according to BMI, while 13 were obese according to PBF. Five subjects had insufficient PSMM.

As shown in Table 3, Pearson’s linear correlation demonstrated small positive correlations between BMI and PBF (r = 0.361, *p* < 0.001), while for BMI and PSMM a small negative correlation was found (r = −0.344, *p* < 0.001). 

**Figure 1:**
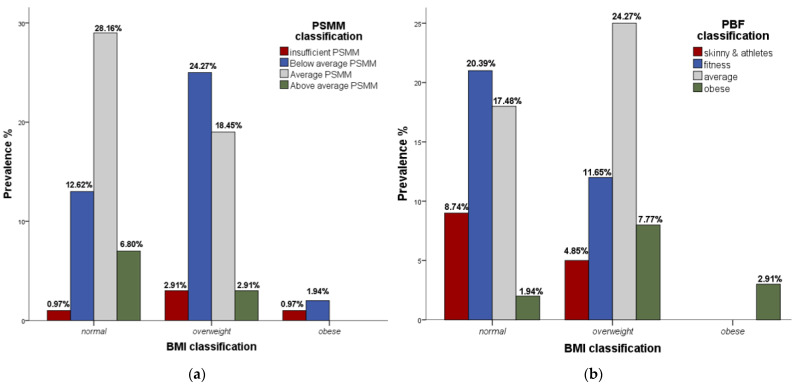
(**a**) PSMM classification within the BMI classification; (**b**) PBF classification within the BMI classification.

Figure 1a revealed certain discrepancies between PSMM and BMI classification (ꭓ^2^ = 29.124, *p* < 0.001). Namely, in the BMI normal group, 13 subjects were classified with below average PSMM, while there was one subject with insufficient PSMM and still classified as normal according to BMI. On the other hand, in the BMI overweight group, three subjects were classified with above average PSMM and 19 subjects with average PSMM. Similar results are shown in Figure 1b (ꭓ^2^ = 15.845, *p* = 0.015), where two subjects were obese according to PBF and still classified as normal according to BMI. In addition, 12 subjects who were categorized for PBF classification as fitness and five who were classified as skinny and athletes were in the BMI overweight group. There were no discrepancies in obese BMI group in both figures. These above-mentioned results are specifically drawn from Figures and highlighted due to discrepancies between the BMI, PSMM and PBF classifications. 

## 4. Discussion

The primary goal of this study was to compare obesity status via the indirect measure of BMI with the direct measure of PBF in PTs and to determine how BMI classifies subjects who possess different levels of PSMM. The main findings of this study showed that there are key differences in the classification of subjects and that it is desirable to use the classification of subjects based on the PBF and PSMM classification rather than exclusively based on the BMI classification (Figure 1a,b. Using BMI classification, the prevalence of obesity was low (2.9%), while the prevalence of overweight was higher 48.5%. On the other hand, using PBF classification the prevalence of obesity was 12.6%. PSMM categories classified 4.9% of subjects with insufficient and 38.8% with below average muscle mass. These results reflect how BMI can mis-classify obese subjects as overweight (*n* = 8) or even normal weight (*n* = 2), while those with greater muscle mass were categorized as overweight (*n* = 3).

Obesity is defined as the accumulation of fat mass in the body, to the point where it could have serious consequences on health [27] and also negatively affect functional movement patterns [31]. As an indirect method of measuring obesity, BMI does not account for the fact that muscle mass decreases with age, while body fat increases, but a person’s weight and height do not necessarily demonstrate the same changes [32]. Although BMI is the least expensive method and most practical measure, it does not seem to be the most ideal indicator of obesity for these PTs or among other samples of POs [2,11,16]. A key reason is that BMI cannot distinguish between fat mass and lean mass in categorizing obesity [33]. We only found a small positive correlation between BMI and PBF and a small negative correlation between BMI and PSMM in this study, indicating that among our sample these measures had only small agreement. 

In this study, 22 subjects who were categorized as overweight by BMI had average or above average PSMM. This is similar to previous research that confirmed subjects with increased muscle mass can be mistakenly classified as overweight [14,15]. Conversely, one subject (1%) with insufficient PSMM was classified in the normal BMI category, while based on the PBF category he was classified in the obese group. This reflects the concept of “skinny fat,” where normal weight individuals actually carry a high relative body fat due to low muscle mass [34]. Although we did not have any subjects who were underweight based on BMI. Kukic et al. [21] found that POs with normal PSMM were classified as such and conclude that BMI classification may be misleading in regard to potential for performance, which is highly important for tactical athlete populations.

The relationship of BMI and PBF classification showed some misclassification of PTs. Inspection of Figure 1b reveals that 11.7% of subjects who were in the fitness PBF group and 4.9% who were in the skinny and athletes PBF group were classified as overweight according to BMI classification. On the other hand, 1.9% of subjects from the obese PBF group were still classified as normal weight according to BMI. Similarly, Heinrich et al. [2] found that BMI was less accurate when compared with PBF in defining obesity among Russian POs. They concluded that it could be more helpful to directly measure PBF, rather than using BMI as a measure of obesity.

### 4.1. Study Limitations

There are a few considerations to discuss for this study. For example, while this study provides helpful information about males, female PTs should be studied in future research. Results from this study may not extrapolate to older policemen, as they are likely to experience weight gain due to age, poor diet, and less physical activity. In addition, future research should compare obesity and body composition among the general population with PTs or POs, as well as the comparison of the body composition of PTs from different years of study. This study used a cross-sectional design with direct measurements, yet a longitudinal design would allow for tracking changes in PTs over time. Additionally, measuring waist circumference would allow additional insight into the distribution of body fat for the subjects. 

### 4.2. Practical Implications

Data from this study are specific to our sample yet may be compared with findings for other tactical athletes. The results and information provided by this research can be used to help professionals understand the importance of measuring body composition, and the negative aspects of BMI classification. Measuring body composition can give both sports experts and respondents an insight into their health and physical condition and help facilitate planning for physical training and nutrition programs. 

## 5. Conclusions

In conclusion, the findings of this study suggest that, while BMI can be used as quick assessment of PT’s obesity status, it is not considered to be an entirely accurate method for obesity screening in PTs when compared with measurement of PBF and PSMM. The results of this study indicate that muscular subjects could be misclassified as overweight based on their muscle mass and that PBF identified more subjects as obese. Furthermore, “skinny fat” individuals with a low level of PSMM can be classified into the normal BMI group, even though they have excessive PBF. This is especially important in the tactical athlete population given that they must always be physically ready to perform, sometimes highly demanding, professional duties. Therefore, the use of BMI together with other anthropometric measures is strongly recommended among PTs, as well as other tactical athletes. 

## Figures and Tables

**Table 1:** Descriptive statistics for participant age and body composition (N = 103).

Variables	Calculations	Mean	SD	Min	Max	cV%	K-S Test
Age (years)		21.5	0.6	21.0	23.0		
BH (cm)		174.1	6.3	147.0	190.0	3.63	0.200
BM (kg)		76.0	8.1	58.7	99.2	10.66	0.151
BMI (kg/m^2^)	BM/BH^2^	25.1	2.1	20.9	34.3	8.47	0.002
SMM (kg)		34.9	4.1	24.3	46.3	11.72	0.052
PSMM (%)	(SMM/BM) × 100	46.0	3.2	32.8	52.0	6.85	0.200
BF (kg)		14.7	4.6	6.7	30.4	31.16	0.106
PBF (%)	(BFM/BM) × 100	19.2	5.2	9.5	41.1	26.81	0.200

BH—body height, BM—body mass, BMI—body mass index, SMM—skeletal muscle mass, PSMM—percent of skeletal muscle mass, BF—body fat, PBF—percent of body fat, K-S Test—Kolmogorov–Smirnov Test, cV—Coefficient of variation, SD—standard deviation.

**Table 2:** Frequency table for body mass index (BMI), percent skeletal muscle mass (PSMM) and percent body fat (PBF).

BMI	N (%)	PSMM	N (%)	PBF	N (%)
Normal	50 (48.5)	Insufficient	5 (4.9)	Skinny and athletes	14 (13.6)
Overweight	50 (48.5)	Below average	40 (38.8)	Fitness	33 (32.0)
Obese	3 (2.9)	Average	48 (46.6)	Average	43 (41.7)
		Above average	10 (9.7)	Obese	13 (12.6)

**Table 3:** Correlation and Chi-square tests between body mass index (BMI)—percent body fat (PBF) and BMI—percent skeletal muscle mass (PSMM) (N = 103).

Variable	*BMI*	
	r	*p*
PBF	0.361	<0.001 **
PSMM	−0.344	<0.001 **

** Significant at *p* < 0.05 by Pearson’s correlation.

## Data Availability

The datasets analyzed during the current study are available from the lead author on request.
